# ‘It Makes You Sit Back and Think Where You Wanna Go’: Veteran experiences in virtual whole health peer‐led groups

**DOI:** 10.1111/hex.13581

**Published:** 2022-08-11

**Authors:** Ekaterina Anderson, Kelly Dvorin, Bella Etingen, Anna M. Barker, Zenith Rai, Abigail N. Herbst, Reagan Mozer, Rodger P. Kingston, Barbara G. Bokhour

**Affiliations:** ^1^ Center for Healthcare Organization and Implementation Research (CHOIR) VA Bedford Healthcare System Bedford Massachusetts USA; ^2^ Department of Population and Quantitative Health Sciences University of Massachusetts Chan Medical School Worcester Massachusetts USA; ^3^ Center of Innovation for Complex Chronic Healthcare (CINCCH) Edward Hines Jr. VA Hospital Hines Illinois USA; ^4^ Department of Mathematical Sciences Bentley University Waltham Massachusetts USA; ^5^ Veteran Engagement in Research Group, Center for Healthcare Organization and Implementation Research (CHOIR) VA Bedford Healthcare System Bedford Massachusetts USA

**Keywords:** health coaching, peer support, qualitative, telehealth, Veterans, well‐being

## Abstract

**Background:**

The Veterans Health Administration (VHA) is building a Whole Health system of care that aspires to empower and equip each Veteran to pursue a personally meaningful vision of health and well‐being. As part of this effort, VHA has developed Taking Charge of My Life and Health (TCMLH), a peer‐led, group‐based programme that seeks to support Veterans in setting and pursuing health and well‐being goals. Prior research showed TCMLH groups to positively impact Veteran outcomes; yet, little is known about Veterans' own experiences and perspectives.

**Methods:**

We completed semi‐structured telephone interviews with 15 Veterans across 8 sites who had participated in TCMLH groups offered by the VHA in the virtual format between Summer 2020 and Fall 2021. Inductive thematic analysis was applied to interview transcripts to generate themes.

**Findings:**

We identified five themes regarding Veterans' experiences with TCMLH: (1) navigating the virtual format; (2) internalizing the value of health engagement; (3) making healthy lifestyle changes; (4) forging social connections; and (5) taking on a more active role in healthcare.

**Conclusion:**

Veterans perceived virtual TCMLH groups as meaningful and beneficial, yet also highlighted several challenges. Their perspectives speak to the need to supplement time‐limited programmes like TCMLH with ongoing, community‐based support. Virtual group‐based well‐being programmes are a promising innovation. Other healthcare systems may draw on VHA's experience while tailoring format and content to the needs of their patient populations.

**Patient or Public Contribution:**

Veterans were involved as evaluation participants. A Veteran consultant, who is a coauthor on this paper, was engaged through the conceptualization of the evaluation, development of data collection materials (interview guide) and writing.

## 1 INTRODUCTION

The Veterans Health Administration (VHA), the largest integrated healthcare system in the United States and key component of the Department of Veterans Affairs (VAs), is in the process of transforming into a Whole Health system of care, which emphasizes promoting the personal health and well‐being of each Veteran rather than treating discrete diseases.^1^, ^2^ This unprecedented transformation was motivated by recognition from organizational leadership that the older—disease‐oriented, reactive and impersonal—model of care has been an exceedingly poor fit for the needs of Veterans, who frequently manage one or more complex chronic conditions while also struggling with service‐related injuries and the challenges of readjusting to civilian life.^3^, ^4^ While VHA's Whole Health transformation is still unfolding, recent studies have demonstrated that Veteran use of Whole Health services is associated with greater engagement in their healthcare and improved self‐management, perceptions of care, life meaning and purpose and perceived stress.^5^, ^6^ As other healthcare organizations in the United States struggle with managing burdensome costs and optimizing patient experiences and outcomes, VHA's Whole Health transformation has attracted growing attention from other healthcare organizations that are looking to transform their own structures and processes and implement a patient‐centred, proactive approach to care.^7^, ^8^

Adopting the Whole Health model, however, may be challenging for front‐line clinicians for a variety of individual‐ and system‐level factors.^5^, ^9^, ^10^ Further, because the Whole Health model is at odds both with the conventional patient–clinician relationship and the traditional military hierarchy, this shift may *also* be difficult for Veterans. To facilitate Veteran comfort with the Whole Health model of care, VHA has designed the ‘Whole Health Pathway’, an entry point to Whole Health wherein Veterans learn about the core concepts of Whole Health and are empowered to actively engage in their own healthcare.

**Figure 1:**
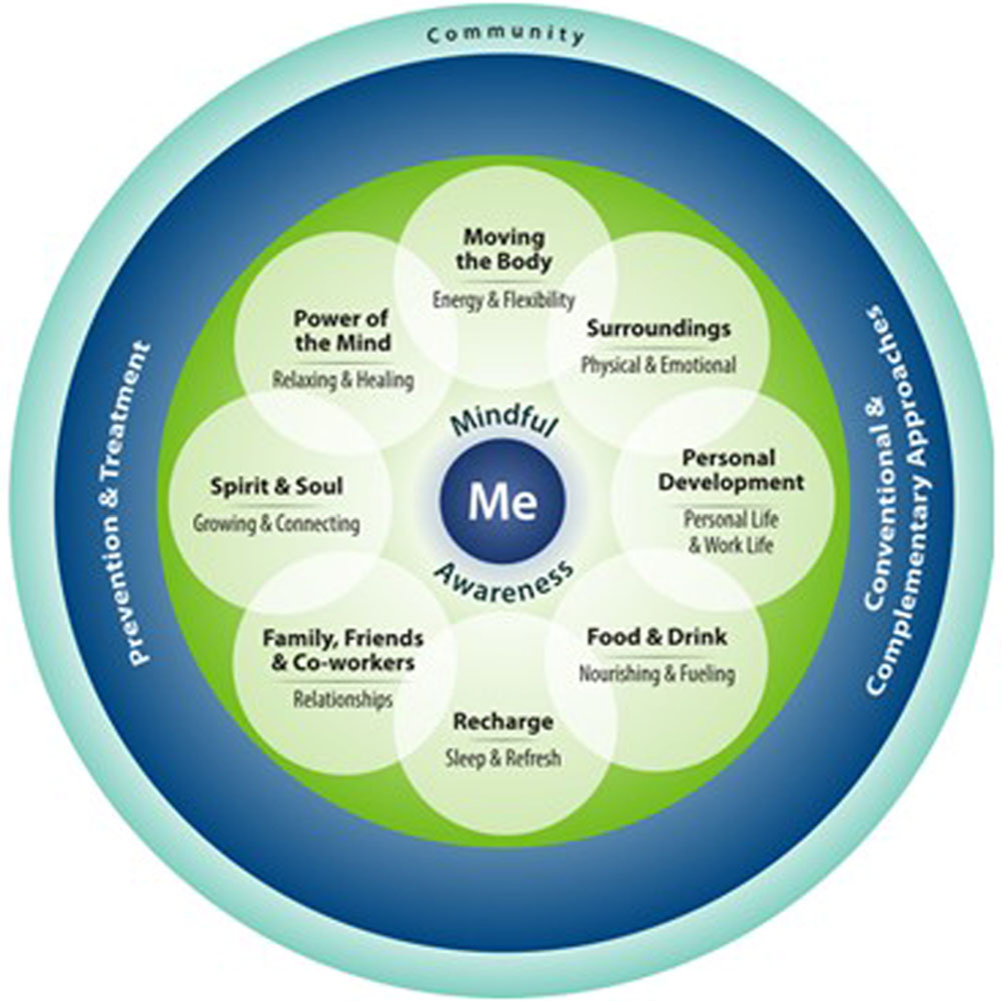
The Circle of Health

While Veterans may choose to enter the Pathway through one‐on‐one meetings with a Whole Health ‘Partner’ (fellow Veteran), VHA also offers a peer‐led, group‐based programme, Taking Charge of My Life and Health (TCMLH), as part of the Pathway. Although the number and length of TCMLH sessions vary across VHA facilities, the key elements remain the same. First, Veterans reflect on their core motivation for becoming more engaged in their health (‘what really matters’) and assess their current experience across eight dimensions of well‐being (e.g., ‘moving the body’, ‘food and drink’, ‘recharge’), presented as the Circle of Health (Figure 1). Throughout the rest of the course, participants explore approaches to enhancing their well‐being across these different dimensions, set SMART (specific, measurable, action‐oriented, realistic and timed) goals^11^ and draw on the group for support and accountability. Ideally, by the end of TCMLH participation, Veterans will have developed self‐care skills, acquired knowledge about well‐being practices that they may want to try (e.g., complementary and integrative health approaches) and gained confidence to take on a more active role in interactions with their healthcare team.

Prior work indicates that TCMLH participation may positively impact Veteran outcomes, including quality of life, sense of meaning in life, mental health and stress, as well as engagement in healthcare and progress towards achieving personal health goals.^12^, ^13^ However, little is known about how Veterans themselves perceive these groups or their impacts. In this paper, we seek to fill this gap by exploring Veterans' perspectives on TCMLH groups.

## 2 METHODS

### 2.1 Overview

Our evaluation of TCMLH, which was part of a larger ongoing effort to evaluate VHA's Whole Health System of Care,^2^, ^14^ took place between Summer 2020 and Fall 2021. It is noteworthy that our evaluation began after VHA facilities across the country had transitioned to the virtual format for the delivery of healthcare and well‐being services, including TCMLH groups, in response to the growing threat of the COVID‐19 pandemic. As a result, all Veterans we recruited had participated in virtual rather than face‐to‐face TCMLH groups. This project was classified as nonresearch/quality improvement by the Institutional Review Board at the VA Bedford Healthcare System.

### 2.2 Participant recruitment

We invited Veterans (*n* = 43) who (1) had taken part in TCMLH and (2) had previously participated in a survey about their experiences in TCMLH, to participate in an interview. We completed interviews with 15 Veterans across 8 VHA sites across the United States (see Table 1).

**Table 1:** Characteristics of study participants

Characteristic	Total
Sex	
Male	12 (80%)
Female	3 (20%)
Race	
White	13 (86.67%)
More than one race	2 (13.33%)
Branch of the military	
Army	6 (40%)
Air force	3 (20%)
Marine corps	1 (6.67%)
Navy	1 (6.67%)
More than one branch	4 (26.66%)
Region	
Northeast	2 (13.33%)
Midwest	5 (33.33%)
South	3 (20%)
West	5 (33.33%)
Length of the TCMLH group offered at site	
6 Sessions	8 (53.33%)
8 Sessions	1 (6.67%)
9 Sessions	5 (33.33%)
Other or unknown	1 (6.67%)
% Of TCMLH sessions attended	
50% or fewer	1 (6.67%)
51%–70%	2 (13.33%)
71%–99%	5 (33.33%)
100%	5 (33.33%)
Unknown	2 (13.33%)
Total	15 (100%)

Abbreviation: TCMLH, Taking Charge of My Life and Health.

### 2.3 Data collection

We conducted semi‐structured telephone interviews between May and July 2021. Both interviewers (E. A. and K. D.) had expertise in qualitative methods. Interviewers followed an interview guide that was developed with input from the full team, including a Veteran consultant (R. P. K.), and included questions about Veterans' experiences with and perspectives about TCMLH (see the Supporting Information Appendix). All interviews were audio‐recorded and transcribed verbatim.

### 2.4 Data analysis

Our analysis was guided by a thematic analysis approach, a widespread, flexible method for generating a rich description of patterns in a data set without following an a priori theoretical framework.^15^, ^16^ We used a combination of deductive and inductive coding. Drawing on a codebook that captured the main domains of the interview guide (e.g., ‘curriculum’, ‘social dynamics’, ‘perceived outcomes’), coders (E. A., K. D. and Z. R.) coded the same set of three transcripts independently. After meeting to resolve discrepancies in code application and introduce additional codes that reflected emerging concepts (e.g., ‘taking responsibility’), coders worked on the remaining transcripts independently. After coding was completed, the team worked to identify and describe overarching patterns and variations across the data set. NVivo qualitative data analysis software (version 12) was used to facilitate coding and analysis.^17^

## 3 FINDINGS

We identified five main themes in Veterans' accounts of TCMLH: (1) navigating the virtual format; (2) internalizing the value of health engagement; (3) making healthy lifestyle changes; (4) forging social connections; and (5) taking on a more active role in healthcare. Themes are described in detail below, with illustrative quotations provided.

### 3.1 Navigating the virtual format

All Veterans we interviewed participated in TCMLH via VHA's Video Connect platform, a format that VHA rapidly embraced in the wake of the COVID‐19 pandemic.^18^ Despite some disruptive technical issues, Veterans generally appreciated the virtual format. Indeed, approximately half of our participants said that they would have preferred a virtual group even if an in‐person option were available and infection control were no longer a concern, citing the accessibility and convenience of virtual groups:

I live really close. It's not a commute down here, it's a parking thing and, honestly, you go up there and you park, and it takes forever. It's a big campus at the VA. Man, you just eliminate all of that, it was like wow. I'll take it. Participant_12_Site_07

Many Veterans also felt that virtual participation fostered a sense of psychological comfort that would have been less possible in person:

I think it made us… more relaxed. …if you're sitting in a conference room or… at a VA hospital trying to do the same thing it feels more closed in, you know, whereas this is… a relaxed setting where you're all just like sitting around… at everybody's houses at the same time. Participant_15_Site_05

Some Veterans did note that the virtual format impeded human connection. One participant attributed this to the difficulty of reading nonverbal cues:

I think there are so many physical cues that I know I give, and… that I've seen… in groups where people are fidgeting, or you can tell when they're either anxious or scared or nervous, and I think a lot of that gets lost in the video. Participant_12_Site_07

Several interviewees also mentioned that the virtual format made it harder to establish informal conversations or to exchange contact information for staying in touch after the programme is completed. A few participants suggested that facilitators should purposefully build in opportunities for socializing:

…you miss some of that, you know…if you're comin' into a room and everybody's gatherin' up in the room and gettin' ready…and there's some…social interchange that occurs prior tothe session. <…> …so, I think, it's very important…if you're gonna do an hour‐long session, add 10 minutes or 30 minutes, so that there is a little bit of social interaction in front of and behind the session. Participant_04_Site_09

### 3.2 Internalizing the value of health engagement

Across our data set, interviewees repeatedly referenced internalizing the value of health engagement, that is, becoming convinced that pursuing greater health and well‐being is a feasible and worthwhile aspiration. The importance of self‐care, framed by one interviewee as ‘being good to yourself’, was especially prominent:

That's the common theme there is to be really good to yourself and that has been an eye‐opener for me because… I've spent… more time in my life beating myself up than I have been being good to myself and finally it's taken hold… <…> …the idea of being good to yourself, such a simple statement, and… now you're able to open up and actually do it…<…> …knowing that these other people were going through the same thing, it just clicked. <…> The kids are gone, I'm retired. For the love of God, do it before the rest of your life goes by. Participant_09_Site_04

Interviewees noted that TCMLH created a motivation to engage in specific practices that promote health and well‐being. As one interviewee fondly recalled:

…by the end of the class people were talking about, ‘Hey, I wanna lose weight, I wanna…stop smoking, I wanna start going for walks. I wanna start meditating more. I wanna start seeing my kid, my grandkids more’. Whatever it was it seemed like people were more… in tune with themselves… <…> …it was nice to see those people, you know, re‐engaged…. Participant_12_Site_07

Several elements of the TCMLH curriculum were mentioned as facilitating these attitude shifts. Many interviewees echoed the group's name in invoking how they internalized the idea of ‘taking control’ or ‘taking charge’:

As a Veteran you need to take part, take charge. I guess the way I look at it is be engaged. Just like work or a project…if you really want to be successful, you need to be engaged and… I think a lot of people don't get that. <…> It was put to me that way and…I was like, they're right. It is up to *me*; it is *my* health…. Participant_13_Site_05

Numerous participants mentioned the perspective‐shifting effects of contemplating their well‐being in the context of the Circle of Health (Figure 1), a graphic that is introduced at the beginning of TCMLH and recurs throughout the curriculum:

…your health and wellness goes well beyond just… your body. You know, it's your relationships, it's the things you eat, it's your diet, it's your mental attitude and capability and things of that nature. …I think it's all very important and… people need to understand how it's connected. Participant_04_Site_09

Finally, several interviewees noted the value of learning to set SMART goals, which allowed them to operationalize large‐scale, ambitious and otherwise overwhelming wellness‐related goals as a sequence of manageable and discrete actions:

…it makes you sit back and <think> where do you wanna go and how do you wanna get there, and you take smaller steps rather than bigger steps and you take those smaller steps and you break it down even more. …So that everything is not so overwhelming for you. Participant_10_Site_04

### 3.3 Making healthy lifestyle changes

Beyond increased *commitment* to a healthier lifestyle, some interviewees shared that TCMLH participation spurred them to make changes in their lifestyle and habits, most commonly by eating better and engaging in physical exercise. One participant expressed pride with sticking to healthy eating habits:

…one of the main changes <for> me in that group was to help my own self to take care of myself better… <…> I believed in diet for years but I never <paid> attention. But when I was in the group, they… said, ‘Well, if you're trying to lose weight, make a plan’, and, you know, ‘try to not eat dinner after 6 o'clock, try to eat before that…’ And I was a person that… would eat any time of the day, any time of night. <…> And that change of habit, I get that from the group. Participant_01_Site_01

Another participant shared being able to reduce use of unwanted medications, which they attributed to holistic nutrition ideas that they gained from TCMLH:

…when I started, I was on a lot of medications, okay? <…> …I was takin' all these and they were messin' with my mind. …what <TCMLH> taught me was… foods that I could eat that would help me relax. …I do the teas now… for my health and stuff. So, in doing so, I was able to get off of <pain medication> and I was able to get off of <sleep medication> to sleep. Participant_03_Site_09

Several interviewees also noted that they started engaging in mindfulness and other meditative/reflective practices following TCMLH participation:

…one of the guys… encouraged me to do a gratitude journal, and I do a journal and I also keep track of my pain and my drinking. All of these things… I'm not denying them <but> assessing and working on <them>. <…> I like the idea of the gratitude because you can get so wrapped up in the pain, the troubles, misfortunes, all of it. Participant_11_Site_03

Importantly, several interviewees also mentioned experiencing barriers to acquiring or maintaining healthy habits after TCMLH ended:

…the goal setting for different things…worked pretty well for me and I tried to maintain that afterwards, after the classes and it was helpful. But it did seem to fall off. Participant_13_Site_05

There were some things that I probably should have integrated into my life, but I wound up not… doing them… …like mindful awareness. The idea of being present, that to me is just foreign, you know for some reason. Participant_07_Site_11

### 3.4 Forging social connections

Many interviewees pointed to social connections—with other TCMLH participants and their existing social circle alike—as a major component of the group's positive impact. Numerous Veterans mentioned enjoying the experience of interacting with others on a personal level, with some explicitly commenting that being with other Veterans was preferable to being with nonVeteran civilians:

…a lot of times, I just don't feel like civilians… understand a lot of things. I've been associated with military most of my life… so here is an online class with all these people that did understand and you're… with a group of people that know <you> and <that> you have connection with. Participant_09_Site_04

Another well‐received facet of the social dynamics of TCMLH was the opportunity to share experiences and insights with other participants. Veterans expressed appreciation for the opportunity to learn *about* and *from* others' experiences:

…that's where I think the group format comes in because you hear other people… rattle them off and it's like, you know, it gives you ideas and it gives you, ‘hey, maybe I can shoot for that, too’ or ‘hey, I like that idea’…. Participant_12_Site_07

Finally, Veterans appreciated having an environment that provided both support and accountability. Several interviewees felt that fellow TCMLH participants helped them stay on track and maintain progress towards their goals:

If someone would ask me if I'm doing <my goals>. …one of my things was making sure I preplan my meals, so it was like Saturday and I knew I had to talk to somebody on Monday to make sure I preplanned my meals so I would make sure on Sunday that I could so if they ask me, I wasn't going to lie. So, I made sure that I… did prepare my meals. So, the accountability, just wanting to be included in the group so <they> know that I did my goal… it helped that way. Participant_14_Site_05

Several Veterans also reported staying in touch with other TCMLH participants, and one in particular described a close‐knit group that formed after TCMLH was over:

…I go to the Y with a few friends that was in the group… and now I feel… comfortable with all the other veterans and <this> makes me feel good that I know I'm helping them out and they're helping me out too. Participant_01_Site_01

More frequently, interviewees talked about TCMLH as an impetus to spending more time with family, re‐engage with old acquaintances or taking on a more active role in their communities. One interviewee described teaching the breathing techniques that they learned in TCMLH to their father, which they felt was a bonding experience. Another shared becoming more comfortable interacting with men in social situations, overcoming the wariness that was caused by negative experiences during military service:

…And now, <I'm>… getting into the groups… that have men in there, cause I'm gonna tell you [laughing], I wouldn't have never got into anything like that, you know. And… I would've been so skeptical about talking. But… it's a good thing now. And I can talk to them, and I can joke with the men that are there, you know? Participant_03_Site_09

### 3.5 Taking on an active role in healthcare

Finally, several Veterans described changing how they approached their healthcare following TCMLH participation. For some, this involved exploring new healthcare services or modalities, such as signing up for other group‐based classes or seeking out complimentary and integrative health programmes:

…[the facilitators] referred me to acupuncture, which I loved. And… I'll probably get into chiropractic, too, as soon as I go to my doctor. Participant_14_Site_05

Others pursued one‐on‐one coaching to continue working on their wellness goals outside of TCMLH:

…when I saw the opportunity for a health coach, it reminded me of one‐on‐one sessions I used to do with a psychologist years ago… I said, ‘No, I'm tired of talk therapy’. <…> But… this one‐on‐one with a coach, it's not the same thing. <…> …it's still up to you to do the work, they're just standing on the side and giving suggestions and encouraging you to express yourself about things and how you feel… and I like that coaching thing. Participant_09_Site_04

Some interviewees acknowledged that participating in TCMLH shifted how they interacted with their established healthcare team members. Several Veterans noted that they mentioned TCMLH to their healthcare provider and received a positive response:

They're positive about that. It's good that I'm able to, you know, do that and they liked the idea that, you know, at least I'm trying. Right? Take charge a little bit, you know. Participant_13_Site_05

Several other participants reported more concrete changes. One described learning about the importance of preparing for a healthcare visit in TCMLH and mentioned learning to prepare for encounters by writing down questions for the provider ahead of time. Another interviewee discussed improvements in their ability to advocate for themselves:

…it made me a better patient for one thing. <…> …I don't get into that resentment area. <…> They're like, ‘Oh well, you need an MRI. Well, we can't do it for six weeks…’ …and I didn't stay satisfied with the first appointment. … But not from a place of resentment, more from a place of – the sooner we know what's going on the better my health will be, so… I got in contact with the people that schedule the MRIs… and I said, ‘I'll go anywhere, if you could just find me the soonest one, I appreciate it’, and they did. So, it made it like three weeks instead of six. Participant_06_Site_11

## 4 DISCUSSION

Our findings demonstrate the potential impact of a patient‐centred, peer‐led group focused on engaging patients in reflection and action around what matters most to them. This study yields insights regarding system‐level changes around implementing patient‐centred care that may be leveraged in both research and practice settings. First, whereas previous survey findings have demonstrated that Veterans may be receptive to and derive benefits from VHA's peer‐led, group‐based TCMLH programme,^13^, ^19^ our current results provide in‐depth elaboration on how Veterans perceive the programme's benefits. This combination of survey data and narratives provides a holistic picture of how and why Veterans derive benefits from TCMLH, information that, when taken together, may be of considerable interest to other healthcare organizations that may be considering the implementation of TCMLH‐style groups as a way to introduce patients to the principles of patient‐centred, empowering, holistic care. Second, we show that Veterans who participated in TCMLH in a virtual format during the COVID‐19 pandemic saw the group as a meaningful and impactful experience, which is an encouraging finding for other healthcare settings that may be interested in implementing TCMLH‐style groups, yet are unsure about how feasible and desirable the virtual format may be to patients. Finally, in what follows, we present the lessons learned and recommendations for other healthcare systems that extend beyond the VHA‐specific context of our current work.

Our key finding was that Veterans who participated in TCMLH achieved precisely the kind of attitude shift that TCMLH was designed to achieve: they internalized the value of health engagement, that is, actively pursuing better health and well‐being.^20^, ^21^, ^22^ In fact, it might be more appropriate to speak of *two* distinct values that were communicated through the programme. First is the importance of self‐care, which, for some participants, may have challenged the stoic values internalized during military service^23^, ^24^, ^25^ and/or other life experiences. Second is the idea of ‘taking charge’ of one's health and healthcare, which invokes the value of self‐reliance *and* subversively reframes it as enthusiastic engagement in, rather than stoic avoidance of, care.^26^, ^27^, ^28^ In other words, TCMLH appears to have promoted *several* attitude shifts in its participants, challenging some values that Veterans held and reframing others. We recommend that healthcare systems seeking to implement TCMLH‐style groups tailor the content and style of such groups in a way that acknowledges the historically and culturally specific meaning(s) of engaging in one's health common among the patient population(s) they serve.

Another key finding is that, for some individuals, TCMLH may serve as a gateway to a healthier lifestyle, in effect working as a quasi‐coaching intervention. Given that more than half of our interviewees participated in a shorter, six‐session version of TCMLH (Table 1), this is very encouraging. It is, perhaps, even more encouraging in light of the fact that we interviewed Veterans who had participated in virtual TCMLH in the early stages of the COVID‐19 pandemic, i.e., following a rapid implementation and before sites had an opportunity to identify or integrate best practices. However, while well‐being groups like TCMLH may hold great promise, they also have significant limitations if offered as a stand‐alone programme. As is well documented in the literature,^29^, ^30^, ^31^ maintaining healthy behaviours over time is challenging, and it is not surprising that some interviewees reported difficulties sticking with their goals after the group ended. It is essential that participants in TCMLH and similar programmes are provided with resources and ongoing support that would allow them to maintain the momentum, such as access to one‐on‐one health coaching or, indeed, an ongoing well‐being group.

Yet another prominent theme in our findings is the importance of the social dimension of TCMLH. Despite the limitations of the virtual platform, Veterans derived benefits from the relationships that they forged with other TCMLH participants. In some cases, the social connections served as a conduit for the knowledge and skills that the group sought to instil. It is also important to note that the interactions and relationships at the heart of TCMLH might have provided a much‐needed lifeline to Veterans who were socially isolated during early days of the pandemic.^32^ All of these findings are consistent with the larger literature that showcases the benefits of peers as health coaches and patient navigators for health engagement,^33^, ^34^, ^35^ as well as the power of peer support to counteract social isolation, including in the times of the COVID‐19 pandemic.^36^ Therefore, it is important for healthcare systems looking to implement well‐being group programmes akin to TCMLH to create environments that foster an atmosphere of trust and bonding, as well as to consider how the supportive connections established during the group may be maintained after it ends.

Finally, even though the discourse of ‘taking charge’, prominent in the group's curriculum and present in its name, may resonate strongly with some participants, it also echoes a neoliberal ideal of the autonomous, efficient, self‐managing individual.^37^, ^38^ Uncritically encouraging participants to conform to such an ideal in the absence of substantive systemic changes would be misguided. One requisite change is cultural transformation within healthcare organizations to foster greater partnership and patient‐centredness in patient–clinician interactions—a direction where VHA is leading the way with its Whole Health initiative. More broadly, however, the health and well‐being of individuals are inextricably linked to the state of their communities.^39^, ^40^ VHA is increasingly spearheading efforts to address unmet socioeconomic needs and reduce class‐, race‐, gender‐based and other disparities among Veterans,^41^, ^42^ which is an encouraging development. However, other healthcare systems and policymakers must also act to address structural problems that undergird poor health outcomes.

Our evaluation has limitations. The Veterans we interviewed are a self‐selected group of individuals, many of whom had strongly positive and meaningful experiences with the group; it is possible that those who did not respond to our interview requests had less positive experiences with the programme. Additionally, Veteran experiences with and perspectives about TCMLH may differ for those who participate in the groups in person. For some interviewees, a lengthy stretch of time passed between participating in TCMLH and participation in the interview, which may have hampered the recall of relevant experiences. Finally, we recognize that our sample size was limited; however, our sample of 15 Veterans exceeds standards established in the literature, which indicate that 12 or fewer interviews may be sufficient to identify a robust range of themes pertinent to the area of interest, especially when the interviewee group is relatively homogeneous, the topic of inquiry specific is rather than vague, the interview technique is of high quality and the interviewees are knowledgeable about the topic.^43^

## 5 CONCLUSION

Veterans describe TCMLH, a peer‐led well‐being group offered by VHA as part of its emerging Whole Health system of care, to be a meaningful and worthwhile experience, with the virtual format offering both challenges and distinct benefits. Veterans' accounts of the group's positive impacts speak to the promise of programmes that integrate peer support and health coaching approaches to promote health engagement, whereas their struggles highlight the need for ongoing, community‐based support beyond the confines of time‐limited programmes.

Our findings carry implications not only for the VHA but also for other healthcare organizations that may be interested in implementing novel approaches to empowering and supporting patients in the pursuit of health and well‐being. While TCMLH is a VHA group targeting Veterans, it integrates broadly applicable principles and thus can serve as a promising framework for empowering patients to pursue greater health and well‐being, as well as a way to introduce patients to the principles of patient‐centred care and patient empowerment. Other healthcare systems may consider using TCMLH as a model for developing and implementing their own peer‐led, group‐based well‐being programmes, tailored to the needs and characteristics of the patient populations that they serve. VHA's experience also suggests that offering such programmes in a virtual format may be a feasible and desirable innovation.

## AUTHOR CONTRIBUTIONS

Ekaterina Anderson was responsible for the conceptualization, evaluation design, data collection, data analysis and writing of the original draft. Kelly Dvorin was involved in conceptualization, evaluation design, data collection and data analysis. Bella Etingen was involved in conceptualization and evaluation design. Anna M. Barker was involved in conceptualization, project administration, evaluation design and data curation. Zenith Rai was involved in data collection, visualization and data analysis. Abigail N. Herbst was involved in evaluation design and data curation. Reagan Mozer was involved in study design. Rodger P. Kingston (Veteran consultant) was involved in conceptualization, evaluation design and data analysis. The senior author (Barbara G. Bokhour) was responsible for funding acquisition, supervision, conceptualization and evaluation design. All authors were involved in the review and editing of the manuscript drafts, have approved the final version to be published and agreed to be accountable for all aspects of the work.

## CONFLICT OF INTEREST

The authors declare no conflict of interest.

## ETHICS STATEMENT

All data were collected and processed in line with Veterans Affairs (VAs) guidelines for nonresearch/quality improvement projects. The nonresearch determination was made by the Institutional Review Board at the VA Bedford Healthcare System. At initial contact, prospective participants were provided with an information sheet about the project. At the start of each interview, the interviewer reviewed the project's purpose and elements of informed consent. Interviews began only upon answering prospective participants' questions and obtaining their verbal assent. All interviews were recorded with participants' permissions.

## Supporting information

Supplementary information.

## Data Availability

Due to the difficulties of deidentifying qualitative interviews, the full data set cannot be made available to the public.

## References

[1] Krejci LPC K , Gaudet T . Whole health: the vision and implementation of personalized, proactive, patient‐driven health care for veterans. Med Care. 2014;52(12):s5‐s8. 25397823 10.1097/MLR.0000000000000226

[2] Bokhour BG , Haun JN , Hyde J , Charns M , Kligler B . Transforming the veterans affairs to a whole health system of care: time for action and research. Med Care. 2020;58(4):295‐300. 32044866 10.1097/MLR.0000000000001316

[3] Gaudet T , Kligler B . Whole health in the whole system of the veterans administration: how will we know we have reached this future state? J Altern Complement Med. 2019;25(S1):S7‐S11. 30870023 10.1089/acm.2018.29061.gau

[4] Waszak DL , Holmes AM . The unique health needs of post‐9/11 U.S. veterans. Workplace Health Saf. 2017;65(9):430‐444. 28849739 10.1177/2165079916682524

[5] Bokhour B , Hyde J , Zeliadt S , et al. Whole health system of care evaluation—a progress report on outcomes of the WHS Pilot at 18 Flagship Sites. Veterans Health Administration. 2020.

[6] Bokhour BG , Hyde J , Kligler B , et al. From patient outcomes to system change: evaluating the impact of VHA's implementation of the whole health system of care. Health Serv Res. 2022;57(suppl 1):53‐65. 35243621 10.1111/1475-6773.13938 PMC9108226

[7] Kligler B , Hyde J , Gantt C , Bokhour B . The whole health transformation at the Veterans Health Administration: moving from “what's the matter with you?” to “what matters to you?”. Med Care. 2022;60(5):387‐391. 35283434 10.1097/MLR.0000000000001706

[8] Mills PJ , Bushell WC . Returning wholeness to health. Glob Adv Health Med. 2022;11:2164957X221092358. 10.1177/2164957X221092358 PMC8998361 35419212

[9] Howe RJ , Poulin LM , Federman DG . The personal health inventory: current use, perceived barriers, and benefits. Fed Pract. 2017;34(5):23‐26. 30766277 PMC6370438

[10] Dryden EM , Bolton RE , Bokhour BG , et al. Leaning into whole health: sustaining system transformation while supporting patients and employees during COVID‐19. Glob Adv Health Med. 2021;10:21649561211021047. 34104578 10.1177/21649561211021047 PMC8168024

[11] Bovend'Eerdt TJ , Botell RE , Wade DT . Writing SMART rehabilitation goals and achieving goal attainment scaling: a practical guide. Clin Rehabil. 2009;23(4):352‐361. 19237435 10.1177/0269215508101741

[12] Abadi M , Richard B , Shamblen S , et al. Achieving whole health: a preliminary study of TCMLH, a group‐based program promoting self‐care and empowerment among veterans. Health Educ Behav. 2021;49(2): 347‐357. 34018443 10.1177/10901981211011043

[13] Abadi MH , Barker AM , Rao SR , Orner M , Rychener D , Bokhour BG . Examining the impact of a peer‐led group program for veteran engagement and well‐being. J Altern Complement Med. 2021;27(S1):S37‐S44. 33788603 10.1089/acm.2020.0124

[14] Bokhour BG , Fix GM , Mueller NM , et al. How can healthcare organizations implement patient‐centered care? Examining a large‐scale cultural transformation. BMC Health Serv Res. 2018;18(1):168. 29514631 10.1186/s12913-018-2949-5 PMC5842617

[15] Braun V , Clarke V . Using thematic analysis in psychology. Qual Res Psychol. 2006;3(2):77‐101.

[16] Fereday J , Muir‐Cochrane E . Demonstrating rigor using thematic analysis: a hybrid approach of inductive and deductive coding and theme development. Int J Qual Methods. 2006;5(1):80‐92.

[17] QSR International . NVivo. 2018.

[18] Anderson E , Dvorin K , Etingen B , et al. Lessons learned from VHA's rapid implementation of virtual whole health peer‐led groups during the COVID‐19 pandemic: staff perspectives. Glob Adv Health Med. 2022;11:1‐12. 10.1177/21649561211064244 PMC8795823 35106189

[19] Abadi MH , Drake C , Richard BO , et al. An evaluation of the facilitator training to implement ‘Taking Charge of My Life and Health’, a peer‐led group program to promote self‐care and patient empowerment in veteran participants. Patient Educ Couns. 2020;103:2489‐2498. 10.1016/j.pec.2020.06.014 32690397

[20] Clancy CM . Patient engagement in health care. Health Serv Res. 2011;46(2):389‐393. 21371026 10.1111/j.1475-6773.2011.01254.x PMC3064908

[21] Hibbard JH , Mahoney E . Toward a theory of patient and consumer activation. Patient Educ Couns. 2010;78(3):377‐381. 20188505 10.1016/j.pec.2009.12.015

[22] Graffigna G , Barello S . Spotlight on the Patient Health Engagement model (PHE model): a psychosocial theory to understand people's meaningful engagement in their own health care. Patient Prefer Adherence. 2018;12:1261‐1271. 30050288 10.2147/PPA.S145646 PMC6056150

[23] Abraham T , Cheney AM , Curran GM . A Bourdieusian analysis of U.S. military culture ground in the mental help‐seeking literature. Am J Mens Health. 2017;11(5):1358‐1365. 26229053 10.1177/1557988315596037 PMC5675196

[24] Cogan AM , Haines CE , Devore MD . Intersections of US military culture, hegemonic masculinity, and health care among injured male service members. Men Masc. 2021;24(3):468‐482.

[25] Wilson PH . Defining military culture. J Mil Hist. 2008;72(1):11‐41.

[26] Sayer NA , Friedemann‐Sanchez G , Spoont M , et al. A qualitative study of determinants of PTSD treatment initiation in veterans. Psychiatry. 2009;72(3):238‐255. 19821647 10.1521/psyc.2009.72.3.238

[27] Stecker T , Fortney JC , Hamilton F , Ajzen I . An assessment of beliefs about mental health care among veterans who served in Iraq. Psychiatr Serv. 2007;58(10):1358‐1361. 17914017 10.1176/ps.2007.58.10.1358

[28] True G , Rigg KK , Butler A . Understanding barriers to mental health care for recent war veterans through photovoice. Qual Health Res. 2015;25(10):1443‐1455. 25488935 10.1177/1049732314562894

[29] Thorsen IK , Kayser L , Teglgaard Lyk–Jensen H , Rossen S , Ried‐Larsen M , Midtgaard J . “I tried forcing myself to do it, but then it becomes a boring chore”: understanding (dis)engagement in physical activity among individuals with type 2 diabetes using a practice theory approach. Qual Health Res. 2022;32(3):520‐530. 34964675 10.1177/10497323211064598

[30] Middleton KR , Anton SD , Perri MG . Long‐term adherence to health behavior change. Am J Lifestyle Med. 2013;7(6):395‐404. 27547170 10.1177/1559827613488867 PMC4988401

[31] Marcus BH , Dubbert PM , Forsyth LH , et al. Physical activity behavior change: issues in adoption and maintenance. Health Psychol. 2000;19(1S):32‐41. 10709946 10.1037/0278-6133.19.suppl1.32

[32] Purcell N , Sells J , McGrath S , Mehlman H , Bertenthal D , Seal KH . “Then COVID happened…”: veterans' health, wellbeing, and engagement in whole health care during the COVID‐19 pandemic. Glob Adv Health Med. 2021;10:216495612110538. 10.1177/21649561211053828 PMC8842446 35174002

[33] Fisher EB , Coufal MM , Parada H , et al. Peer support in health care and prevention: cultural, organizational, and dissemination issues. Annu Rev Public Health. 2014;35:363‐383. 24387085 10.1146/annurev-publhealth-032013-182450

[34] Heisler M . Different models to mobilize peer support to improve diabetes self‐management and clinical outcomes: evidence, logistics, evaluation considerations and needs for future research. Fam Pract. 2010;27(suppl 1):i23‐i32. 19293400 10.1093/fampra/cmp003 PMC2902359

[35] McBrien KA , Ivers N , Barnieh L , et al. Patient navigators for people with chronic disease: a systematic review. PLoS One. 2018;13(2):e0191980. 29462179 10.1371/journal.pone.0191980 PMC5819768

[36] Suresh R , Alam A , Karkossa Z . Using peer support to strengthen mental health during the COVID‐19 pandemic: a review. Front Psychiatry. 2021;12:714181. 34322045 10.3389/fpsyt.2021.714181 PMC8310946

[37] Lawn S , McMillan J , Pulvirenti M . Chronic condition self‐management: expectations of responsibility. Patient Educ Couns. 2011;84(2):e5‐e8. 20705412 10.1016/j.pec.2010.07.008

[38] Ayo N . Understanding health promotion in a neoliberal climate and the making of health conscious citizens. Crit Public Health. 2012;22(1):99‐105.

[39] Wagemakers A , Vaandrager L , Koelen MA , Saan H , Leeuwis C . Community health promotion: a framework to facilitate and evaluate supportive social environments for health. Eval Program Plann. 2010;33(4):428‐435. 20106527 10.1016/j.evalprogplan.2009.12.008

[40] Goldberg D . Social justice, health inequalities and methodological individualism in US health promotion. Public Health Ethics. 2012;5(2):104‐115.

[41] Duan‐Porter W , Martinson BC , Greer N , et al. Evidence review‐social determinants of health for veterans. J Gen Intern Med. 2018;33(10):1785‐1795. 30030735 10.1007/s11606-018-4566-8 PMC6153229

[42] Sjoberg H , Liu W , Rohs C , et al. Optimizing care coordination to address social determinants of health needs for dual‐use veterans. BMC Health Serv Res. 2022;22(1):59. 35022053 10.1186/s12913-021-07408-x PMC8754195

[43] Guest G , Bunce A , Johnson L . How many interviews are enough? An experiment with data saturation and variability. Field Methods. 2006;18(1):59‐82.

