# Supplementary material for: ‘It Makes You Sit Back and Think Where You Wanna Go’: Veteran experiences in virtual whole health peer‐led groups
Source: Health Expect. 2022 Aug 11;25(5):2548–56. doi: 10.1111/hex.13581 (PMC9615064; doi:10.1111/hex.13581)
Supplement: Supplementary file 1 — Supplementary information. [file HEX-25--s001.docx]

**Appendix: Interview Guide for Veterans**

**Preliminary questions / Background**

Most of our interview will be focused on your experiences with the Taking Charge of My Life and Health group. Before talking about that I wanted to take a few minutes to learn a little about you as a person.

Let’s start with your military service. What branch of the military did you serve in?

**If needed**: When did you serve? For how long?

How long have you been receiving treatment or services at the VA?

What kind of care or services do you typically go to the VA for? [Probes: Types of providers seen; types of treatment or procedures; any broader activities in the VA that are related to health and well-being, e.g. CIH]

Do you receive any healthcare or participate in any well-being activities outside the VA?

1. Can you share a few more things about yourself that would help me get to know you a little better? [Probe if needed: work, family, living situation, hobbies.]

**Joining TCMLH**

Now I have some questions about the time when you joined Taking Charge of My Life and Health, or TCMLH.

How did you become involved in TCMLH?

**If needed**: How did you come to first learn about TCMLH?

If you recall, what expectations or hopes did you have for the group, if any?

1. Could you give me a sense of what your daily life was like around the time when you signed up for TCMLH?

How would you describe your health and well-being around the time you signed up for TCMLH?

1. **If needed:** What are some concerns, if any, that you had related to your health and well-being?

I have a few questions about your attendance of TCMLH groups. From our records, it looks like you were in a TCMLH session that started on [date] and finished on [date].

Can you recall how many weekly meetings of the group did you attend? Did you ever miss a class? I know that life can get so busy, and we all miss appointments from time to time.

[If missed some meetings.] Do you remember which sessions you missed?

Was this your first experience with TCMLH, or did you ever attend this group in person, prior to COVID?

**Experiences in TCMLH**

1. All things considered, how would you describe your experience in TCMLH?
2. I know that you participated in a TCMLH group virtually rather than in person. What was that like for you?
   1. **Probe if needed:** What was challenging about the virtual format, if anything? What did you enjoy about the virtual format, if anything?
   2. **If Veteran previously participated in a face-to-face class**: How does the virtual format compare to in-person?
3. There are so many aspects of TCMLH that we could talk about. Was there anything about the group that particularly stood out for you?

**If nothing comes to mind for this question, consider the following probes:**

- What, if anything, was challenging about the TCMLH group?
- What, if anything, was enjoyable, useful or meaningful about the TCMLH group?
- Was there a moment during the group that was particularly memorable or important to you? Please describe it for me. [If none of these probes resonate, ask about specific things, like MAP / Wheel of Health / SMART goals.]

1. Overall, what was it like to **be in this group with other Veterans**? What was the atmosphere or dynamic in the group like, from your perspective?
2. Tell me about your impression of the **group facilitator**.
   1. **If needed**: What did the facilitator do well? What could have been done better?
3. If you had an option to take TCMLH in person – let’s say if COVID wasn’t a concern – **would you opt for the in-person group**? Why / why not?
4. Have you attended any other groups or group-based classes in VA? [If yes] How does TCMLH **compare to those groups**? Is there anything special about TCMLH?

**Experiences since completing TCMLH**

So, it’s been [**XX months**] since you completed TCMLH. Since taking part in TCMLH, what has your life been like?

**If needed**: Have you noticed changes in your life?

**If needed**: Is there anything you started or stopped doing? Please think broadly.

**If needed**: Have you stayed in touch with anyone from the course? If so, please tell me about it.

Since enrolling in TCMLH, have there been any **changes in the healthcare services** you have used?

**If needed**: Please think broadly. This could be changes in frequency of appointments, seeing a new/different kind of healthcare provider, starting a new treatment or class, or stopping a particular treatment or service.

**If needed**: How about Coaching? Are you or were you working with a health coach at some point?

- 1. **If needed**: What are your main concerns right now, when it comes to your health and well-being?

1. Have you brought up TCMLH and/or something that you learned in it with your PCP or other provider?
   1. **If yes**, what was that like?
   2. **If no**, can you imagine yourself doing so in the future? Why or why not?

**Recruitment and Retention**

From our work we have heard that in some places not many Veterans participate in TCMLH, or they may start the class series, but not complete it. We would like to hear your perspective on this.

1. First, is this something you have you observed or experienced?
2. Why do you think it might be that not many Veterans participate in TCMLH or start it but don’t complete it?
3. What are your recommendations for improving attendance at TCMLH classes?

**Conclusion**

1. All things considered, what can VA do to improve Veterans’ experience with TCMLH groups?
2. Is there anything else you’d like to add, or any questions for our research team?
